# Developmental stage-specific A-to-I editing pattern in the postnatal pineal gland of pigs (*Sus scrofa*)

**DOI:** 10.1186/s40104-020-00495-6

**Published:** 2020-09-07

**Authors:** Rong Zhou, Wenye Yao, Chundi Xie, Leixia Zhang, Yangli Pei, Hua Li, Zheng Feng, Yalan Yang, Kui Li

**Affiliations:** 1 Guangdong Provincial Key Laboratory of Animal Molecular Design and Precise Breeding, Key Laboratory of Animal Molecular Design and Precise Breeding of Guangdong Higher Education Institutes, School of Life Science and Engineering, Foshan University, Foshan, 528231 Guangdong China; 2 State Key Laboratory of Animal Nutrition; Key Laboratory of Animal Genetics Breeding and Reproduction, Ministry of Agriculture and Rural Affairs, Institute of Animal Science, Chinese Academy of Agricultural Sciences, Beijing, 100193 China

**Keywords:** A-to-I, Pig, Pineal gland, Postnatal development, RNA editing

## Abstract

**Background:**

RNA editing is a widespread post-transcriptional modification mechanism in mammalian genomes. Although many editing sites have been identified in domestic pigs (*Sus scrofa*), little is known about the characteristics and dynamic regulation of RNA editing in the pineal gland (PG), a small neuroendocrine gland that synthesizes and secretes melatonin, which is primarily responsible to modulate sleep patterns.

**Results:**

This study analyzed the expression of adenosine-to-inosine (A-to-I) editing regulators and profiled the first dynamic A-to-I RNA editome during postnatal PG development. The results identified *ADAR1* as the most abundantly expressed ADAR enzyme, which was down-regulated during postnatal PG development. Furthermore, 47,284 high-confidence RNA editing sites were identified, the majority of which (93.6%) were of the canonical A-to-I editing type, followed by C-to-T editing. Analysis of its characteristics showed that the A-to-I editing sites mostly localized in SINE retrotransposons PRE-1/Pre0_SS. Moreover, a strong deficiency and preference for guanine nucleotides at positions of one base upstream or downstream were found, respectively. The overall editing level at the puberty stage was higher than at both infancy and adulthood stages. Additionally, genome-wide RNA editing was found to exhibit a dynamic stage-specific fashion (postnatally). Genes that underwent developmental changes in RNA editing were associated with catabolic processes as well as protein localization and transport functions, implying that RNA editing might be responsible for the molecular machineries of the postnatal developing PG. Remarkably, RNA editing in 3′-UTRs might regulate gene expression by influencing miRNA binding during PG development.

**Conclusions:**

This study profiles the first comprehensive developmental RNA editome in the pig PG, which contributes to the understanding of the importance of post-transcriptionally mediated regulation during mammalian postnatal PG development. Moreover, this study widely extends RNA editome resources in mammals.

## Background

RNA editing is a widespread post-transcription modification mechanism in mammalian genomes that alters the nucleotide composition at the RNA level, while no affecting the corresponding DNA sequence [1]. Among different RNA editing types, adenosine-to-inosine (A-to-I) RNA editing is the most prevalent form in mammals, and is catalyzed by adenosine deaminase acting on the RNA (ADAR) protein family. Consequently, during translation, inosine is recognized as guanosine (G) by the cellular machinery during translation. With regard to functional consequences, A-to-I editing at protein-coding regions can result in changes of amino acids [2]. A-to-I editing also plays an important role in gene expression by modulating alternative splicing [3], editing miRNA sequences [4] and affecting miRNA binding sites [5]. For instance, A-to-I editing influences the propagation of fast electrical and chemical signals in nervous systems [6] and is both temporally and developmentally regulated during brain development and diseases [7, 8].

With the rapid adoption of high-throughput sequencing technologies, the RNA editome has been profiled in human and other mammals across many tissues and developmental stages [7, 9–11]. The results identified more than a hundred million RNA editing sites in mammalian genomes and on the majority of genes [12]. Several studies have recently identified and reported the characteristics of RNA editing in the genome of pigs, a major source of meat and an ideal biomedical model [13–16]. For example, the RNA editome in the pig skeletal muscle across 27 developmental stages showed that the overall editing level decreased throughout development and RNA editing is a vital regulator of myogenesis and muscle development [13]. These studies provide rich resources for a better understanding of the functions and mechanisms of RNA editing during various biological processes.

The mammalian pineal gland (PG) is a neuroendocrine transducer whose main, and most conserved, function is the conversion of photoperiodic information into the synthesis and secretion of the nocturnal hormonal signal melatonin [17]. Melatonin exerts critical roles in a number of neuroendocrine and physiological processes in mammals, such as circadian rhythms, vision, reproduction, obesity and cancers [18–21]. Postnatal development of the PG is a highly dynamic period of tissue remodeling and phenotype maintenance. Our previous study provided the first dynamic transcriptome of the porcine postnatal PG [22]. However, dynamic epitranscriptomics in the PG have not been reported for mammals; consequently, the regulation of RNA editing during postnatal PG development remains unclear to date.

This study systematically identified and characterized the RNA editome of developing porcine PGs by combining strand-specific total RNA sequencing and whole-genome re-sequencing data. Most of the RNA editing sites exhibited developmental-dependent changes during postnatal PG development. The differentially edited sites (DESs) in the 3′-UTR were found to regulated gene expression by affecting miRNA binding. The present study profiled the dynamic editome in the pig PG, which provides a rich resource for epitranscriptome studies in pigs and an exceptional opportunity to study PG development in mammals.

## Methods

### Transcriptome data

The transcriptome data, which were generated from the PG of Yorkshire (Y) pigs at postnatal days 30, 180, and 300 (abbreviated as Y30, Y180, and Y300, respectively), were obtained from our previous study (SRA accession number: SRP172576) [22]. The transcriptome data consisted of 1.05 GB strand-specific reads that were sequenced as 150 bp paired-end reads. Each developmental stage used three biological replicates. Clean reads of the nine transcriptomes were mapped to the *Sus scrofa* reference genome using TopHat2 (v2.1.0) [23] with known gene annotation as previously report [22]. The *S. scrofa* reference genome sequence (*Sscrofa* 11.1) and the gene annotation GTF file were downloaded from the Ensembl database (release 90, http://asia.ensembl.org/index.html). Information of long non-coding RNAs (lncRNAs) was obtained from our previous study [22]. The expression levels of each gene were measured as the numbers of reads per kilobase of the exon model in a gene per million mapped reads (RPKM).

### Whole-genome sequencing

Genomic DNA of an individual Yorkshire pig (female) at days 300, which was one of the pigs adopted for RNA-seq analysis, was isolated from the skeletal muscle tissue. The whole-genome sequencing (WGS) library was prepared and sequenced on the Illumina HiSeq X Ten platform (Novogene, Beijing, China) according to the manufacturer’s instructions. A total of 909.92 million paired-end reads (150 bp × 2) were generated from this whole-genome sequencing library, representing a genome-wide coverage depth of ~ 35×. Clean reads were aligned to the reference genome by BWA (v0.7.17). Paired reads were mapped separately using the commands “bwa aln” and “bwa sampe”, allowing a maximum of four mismatches.

### Variant calling

For the aligned bam files of WGS and RNA-Seq, duplicate reads were removed by the MarkDuplicates tool in the Picard package (v2.17.0). Only unique reads (*q* > 10), mapped to the reference genome were retained by samtools (v1.6) [24]. The HaplotypeCaller tool of the Genome Analysis Toolkit (GATK, v3.4) were used to call variants. Variants with a base quality ≥25 and no more than two allele types were retained. The minor allele count of each variant was supported by at least three reads. The variants were annotated by snpEff (v4.3t) based on Ensembl gene annotation (release 90).

### RNA editing detection

The RNA editing sites in PG were identified using a previously published pipeline [13]. This pipeline was modified based on previous studies in other species [9, 10, 12, 25–27] and has been proved to be efficient and solid for the accurately identification of high-confidence RNA editing in pigs [13]. Briefly, the variants called by the transcriptome data were filtered by the following steps: (1) SNPs were discarded that were genotyped as heterozygous variants by WGS and that were present in the dbSNP (v150, ftp://ftp.ensembl.org/pub/release-91/variation/vcf/sus_scrofa/); (2) intronic sites that occurred within 4 bp of splice junctions were removed; (3) variants in homopolymer runs were discarded; (4) variants that located within 6 bp of both ends of a read were filtered to avoid the sequencing errors; (5) Finally, BLAT alignment was used to identify and discard sites in regions with highly similarity to other regions of the genome were discarded. The remaining variants were considered as candidate RNA editing sites.

Only A-to-I editing sites were retained for the following analysis. The overall editing rate of each PG sample was quantified as the ratio of the total number of G reads with at all A-to-I editing positions to all A and G reads covering the editing positions. To obtain the total amount of RNA editing in each PG sample, we took all editing sites we identified into account and did not set any sequencing coverage criteria [9]. The editing level of each RNA editing site was calculated as the number of G reads compared with the total number of A and G reads covering the editing site. To eliminate false positives caused by amplification bias or sequencing errors, at least 10 sequencing reads were required to cover each site with a high-quality score (*q* > 25) and at least three reads were required to support the editing form.

### Identification of differentially edited sites

Significantly differentially edited sites (DESs) between different developmental stages were identified by Student’s *t*-test using cutoffs of FDR ≤ 0.05 and absolute editing differences ≥0.1. Genes that contained at least one DESs were considered as differentially edited genes and were subjected to Gene Ontology (GO) enrichment analysis via the DAVID website (v6.7, http://david.abcc.ncifcrf.gov/) [28].

### miRNA binding site prediction

For the prediction of miRNA binding, two kinds of sequences with regions (50 bp upstream and downstream) flanking the 3′-UTR DESs were prepared first: the reference sequences and A-to-I editing sequences. Then, the miRNA binding sites on the two kinds of sequences were predicted by Miranda software (v3.3a) using default parameters [29]. Mature miRNA sequences of *S. scrofa* were extracted from miRBase (release 22) [30].

## Results

### Expression of ADARs during postnatal pineal gland development

**Fig. 1:**
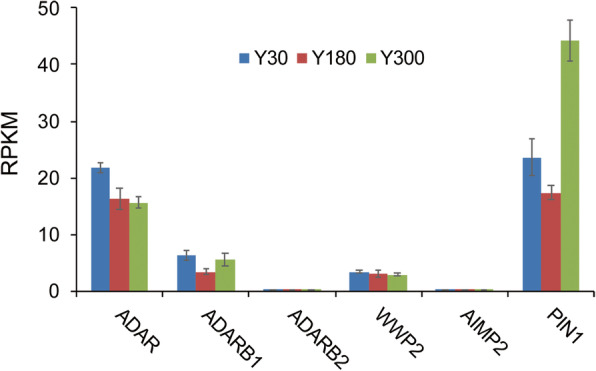
Expression levels of *ADARs* and RNA editing regulators at three developmental stages (Y30, Y180 and Y300) of pig pineal glands. Expression abundance of each gene is measured in reads per kilobase of the exon model in a gene per million mapped reads (RPKM). Error bars are SD across the three biological replicates

First, the temporal expression of ADAR enzymes in postnatal PG was evaluated by RNA-Seq. The results showed that the expression of *ADAR* (also known as *ADAR1*) was higher than that of *ADARB1* (also known as *ADAR2*) and *ADARB2* (also known as *ADAR3*) in postnatal PG and was down-regulated during development. *ADAR2* expression was first down-regulated in Y180 and then up-regulated in Y300, while *ADARB2* was expressed at very low levels (Fig. 1). Furthermore, the expressions of other RNA editing regulators were also evaluated in postnatal PG. *PIN1* and *WWP2* are known to modulate RNA editing by regulating the activity of *ADAR2* through post-translational modification with opposing effects [31]. *PIN1* was found to be abundantly expressed in postnatal PG and its expression trend was similar to that of *ADAR2*. The expression of *WWP2* were decreased throughout development. *AIMP2* negatively regulated RNA editing by decreasing the protein level of ADARs [9]. *AIMP2* was almost not expressed in the postnatal PG (Fig. 1).

### Identification of RNA editing in the pig pineal gland

**Fig. 2:**
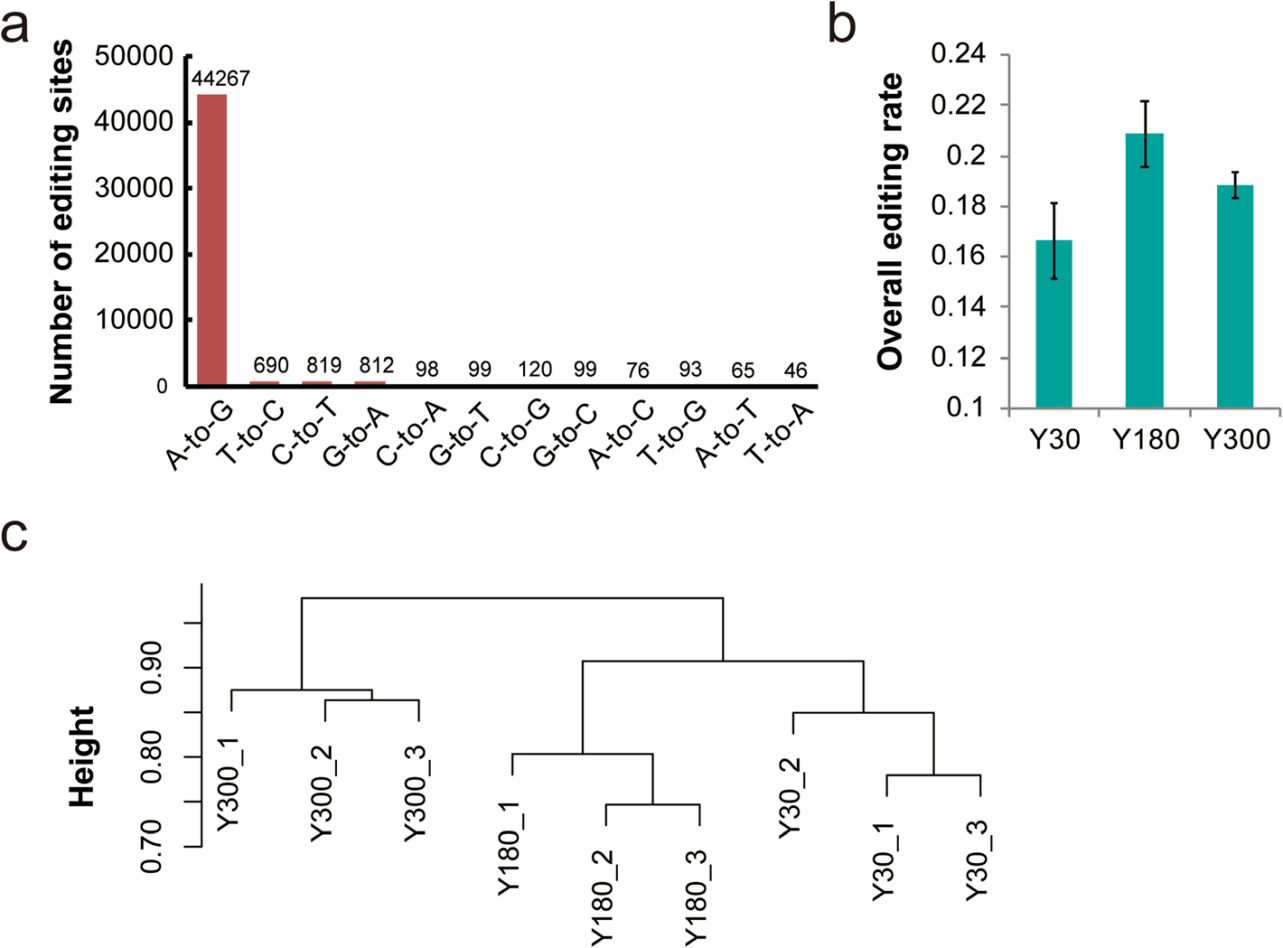
Identification of RNA editing in pig pineal glands. **a** Number of RNA variant types in the postnatal pig PGs. **b** Overall editing level of pig PGs at different developmental stages. **c** Hierarchical clustering analysis of nine PG samples across three developmental stages based on the A-to-I RNA editing level

A total of 47,284 high-confidence RNA editing variants were identified in the pig PG transcriptome using the reported pipeline (see Materials and Methods). As expected, A-to-G (I) editing was the most dominant type of RNA editing (44,267, 93.6% of edits), which was followed by C-to-T editing (1.73%, Fig. 2a). The subsequent analysis focused on the A-to-I editing sites in our s (see Additional File 1). First, the editing level of PGs was evaluated and the overall editing activity at Y180 (20.87%) was found to be higher than those of both Y30 (16.63%) and Y300 (18.86%) stages (Fig. 2b). Clustering analysis, based on the editing level of A-to-I sites, showed that Y30 and Y180 first clustered together and then grouped with Y300, thus reflecting the development-dependent changes of RNA editing during postnatal PG development (Fig. 2c).

### Characteristics of A-to-I editing in the pig pineal gland

**Fig. 3:**
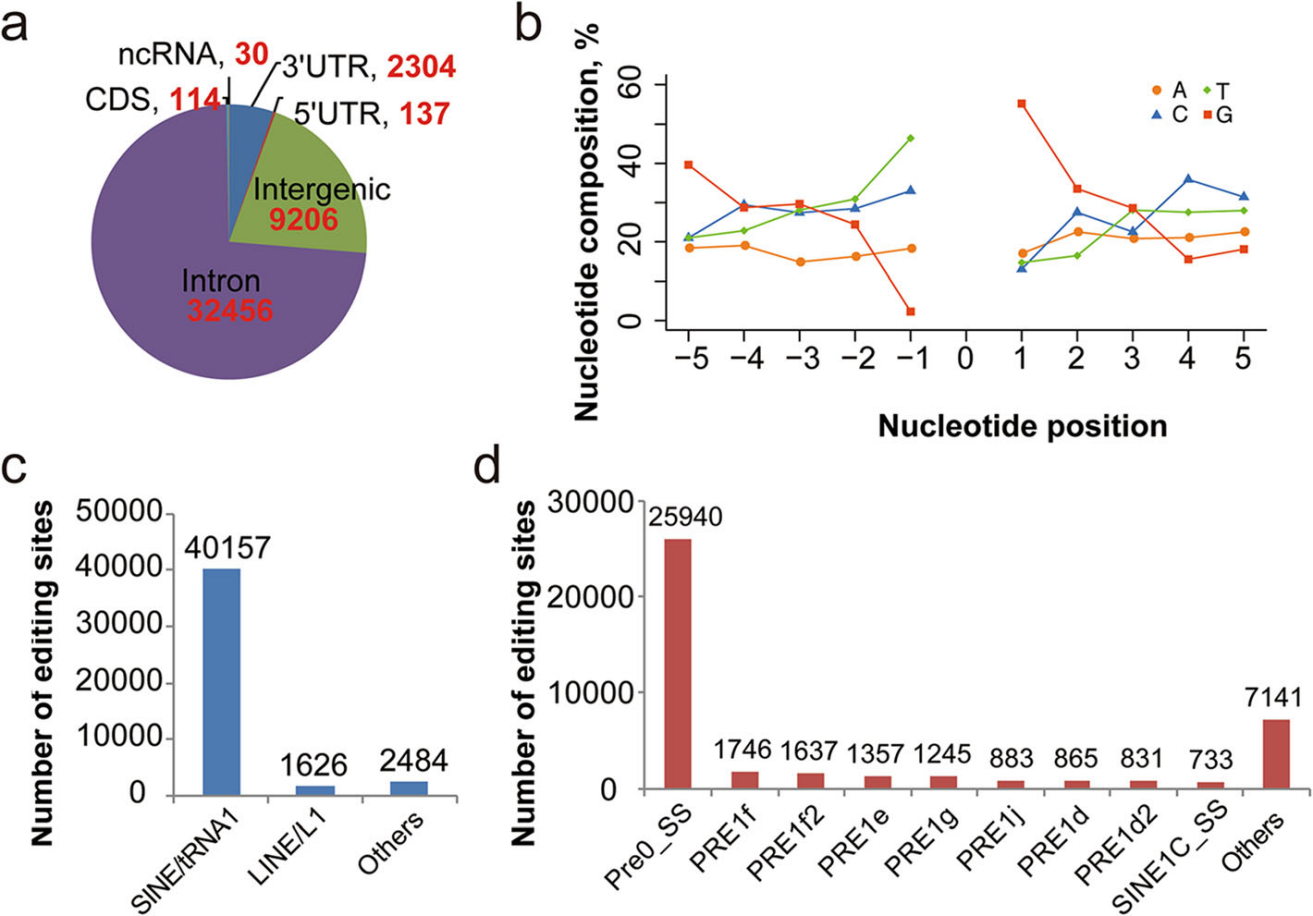
Characterization of the *Sus scrofa* PG editome. **a** Distribution of A-to-I editing sites across different genomic locations. **b** Nucleotide preference flanking the A-to-I editing sites. **c** Distribution of major repetitive element families of A-to-I editing sites. **d** Distribution of A-to-I editing sites across repetitive element types

Gene annotation indicated that most of the editing events occurred in the introns of genes (73.4%), followed by intergenic (20.8%) and 3′-UTR (5.2%) regions (Fig. 3a). 114 of the editing sites overlapped with protein-coding regions (CDS), and 60.5% (69/114) of which led to changes in the encoded amino acids (Fig. 3a). Nearest-neighbor nucleotide analysis of the A-to-I editing sites showed that Gs were enriched one base downstream and depleted one base upstream of the editing sites (Fig. 3b). This matches both the known *ADAR* sequence preferences and the known ADAR targets [32, 33]. As previously reported in other tissues of pigs, A-to-I editing sites are mostly localized in repeat elements (95.7%), most often in SINE/tRNA elements (Fig. 3c). Further analysis showed that the majority of editing sites on SINE/tRNA elements were located within the Pre0_SS (Fig. 3d), a repeat element of the porcine specific SINE retrotransposon PRE-1. Additionally, 699 editing sites were identified in exons of 199 lncRNAs (34 known lincRNA and 165 novel lncRNAs).

### Dynamic RNA editing profiles during postnatal pineal gland development

**Fig. 4:**
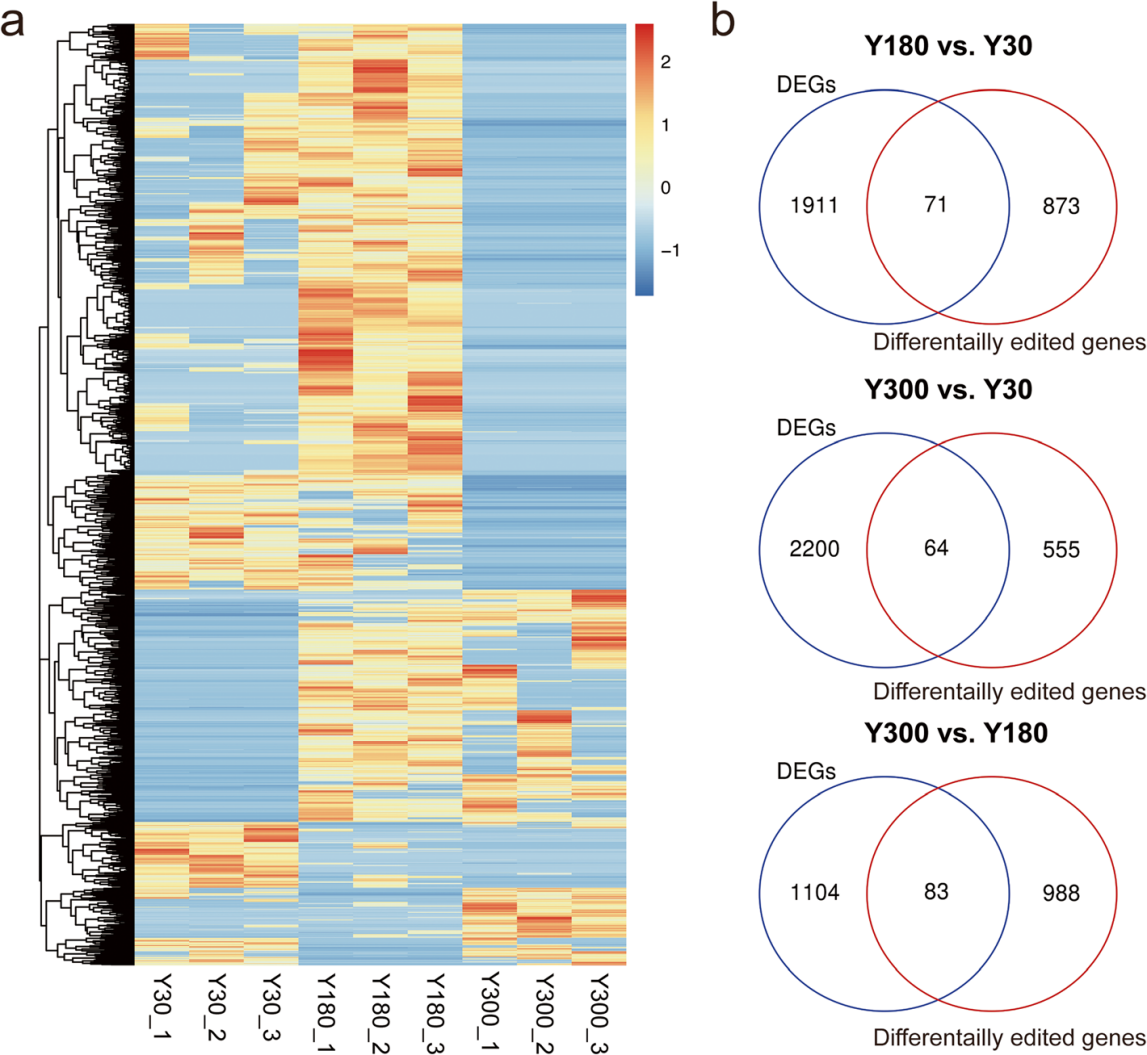
Differential analysis of A-to-I RNA editing during porcine postnatal PG development. **a** Heatmap showing the editing levels of DESs during porcine postnatal PG development. **b** Venn diagram showing the overlaps between differentially expressed genes and differentially edited genes

To explore the dynamic regulation of RNA editing during postnatal PG development, DESs between different developmental stages was identified with significance thresholds of |editing differences| ≥ 0.1 and FDR ≤ 0.05. A total of 3,709 DESs were identified across these three comparisons, including 1,578 Y180-Y30 (1,340 up-regulated and 238 down-regulated), 1,056 Y300-Y30 (335 up-regulated and 721 down-regulated), and 2,135 Y300-Y180 (197 up-regulated and 1,938 down-regulated). Interestingly, no DESs were shared by all the comparisons. A heatmap based on the editing level of all DESs showed that these particular DESs exhibited a stage-specific editing pattern across postnatal PG development (Fig. 4a). Integrative analyses showed that 71, 64, and 83 genes overlapped among the DEGs and differential RNA editing genes in the Y180-Y30, Y300-Y30, and Y300-Y180 comparisons, respectively (Fig. 4b), This includes many genes associated with synaptic transmission and ion transport, such as *CACNB2*, *CACNA1A*, and *CACNA1D*. Remarkably, while there are five genes (*TAF5*, *CEP250*, *DIDO1*, *CXXC4* and *PPP2R3B*) contained DESs in CDS regions that might lead to changes in encoded amino acids, the expressions of these genes did not change significantly during postnatal PG development. For instance, the recoded RNA editing (chr14:114304916) transcription factor TATA-Box binding protein associated factor 5 (*TAF5*) led to an Asn → Ser amino acid substitution. *TAF5* plays a major role in the formation of scaffold that is critical for transcription initiation factor TFIID complex formation [34], implying a potential role in regulating postnatal PG development.

### Functional enrichment analysis of genes with differentially edited sites

**Fig. 5:**
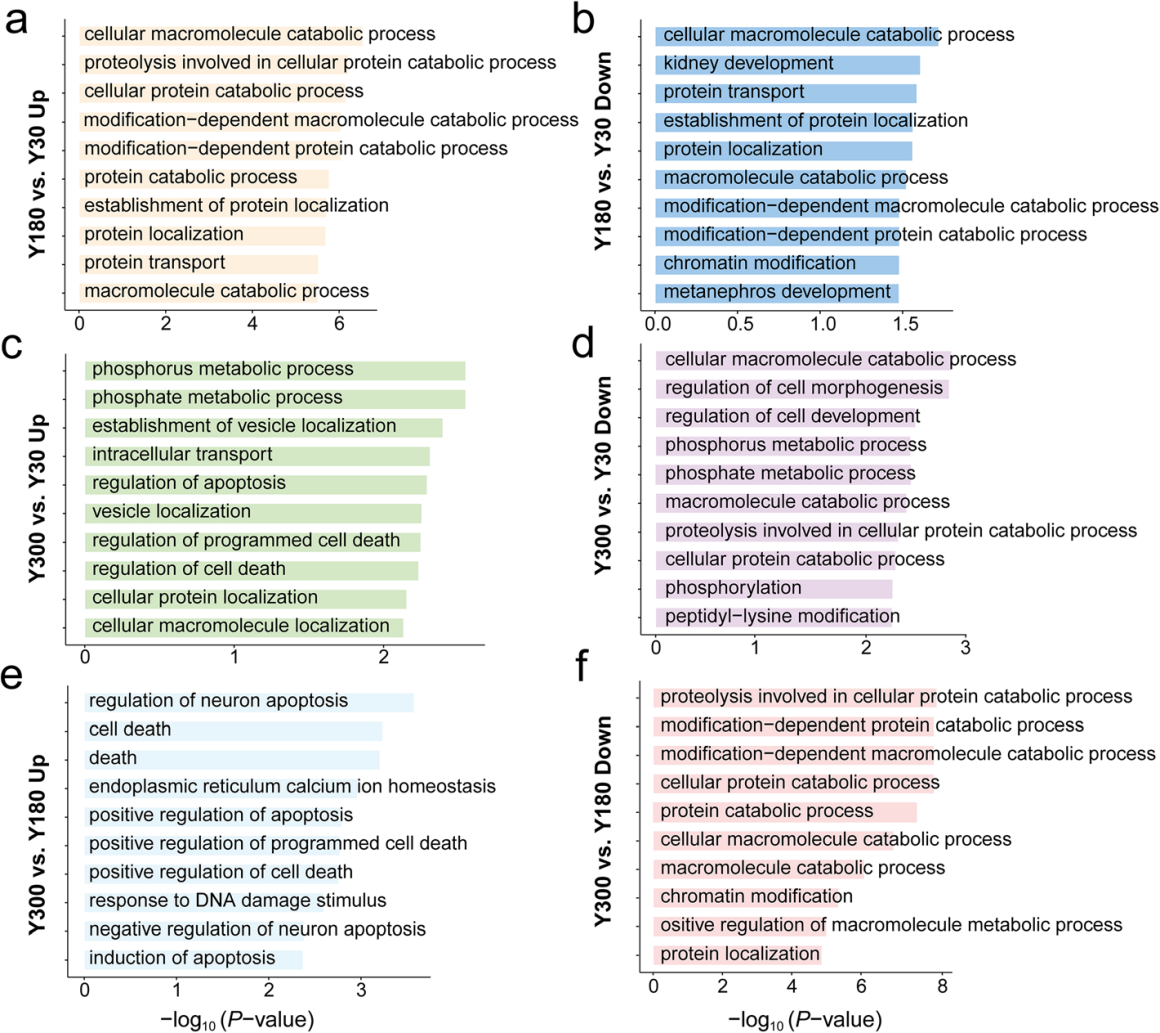
Functional analysis of the differentially editing genes. (**a-b**) Gene Ontology (GO) biological process analysis of the up-regulated (**a**) and down-regulated (**b**) differentially edited genes between Y180 and Y30. (**c-d**) GO biological process analysis of the up-regulated (**c**) and down-regulated (**d**) differentially edited genes between Y300 and Y30. (**e-f**) GO biological process analysis of the up-regulated (**e**) and down-regulated (**f**) differentially edited genes between Y300 and Y180

GO analysis was then performed to examine the enriched biological functions of genes that underwent developmental changes during RNA editing. The results showed that the genes with up-regulated DESs in the Y180-Y30 comparison were enriched in related to catabolism as well as protein localization and transport functions (Fig. 5a, b). Between Y300 and Y30, genes with up-regulated DESs in Y300 were significantly enriched in the phosphate metabolic process, establishment of vesicle localization, intracellular transport functions, and regulation of apoptosis (Fig. 5c). However, genes with down-regulated DESs were associated with catabolic process, phosphorylation, and neuron differentiation functions (Fig. 5d). Between Y300 and Y180, genes with up-regulated DESs in Y300 were significantly enriched in the regulation of neuron apoptosis and cell death functions (Fig. 5e), while genes with down-regulated DESs were significantly enriched in catabolic processes, chromatin modification, as well as protein localization and protein transport functions (Fig. 5f).

### Differentially edited sites in 3′-UTR affected miRNA binding

**Fig. 6:**
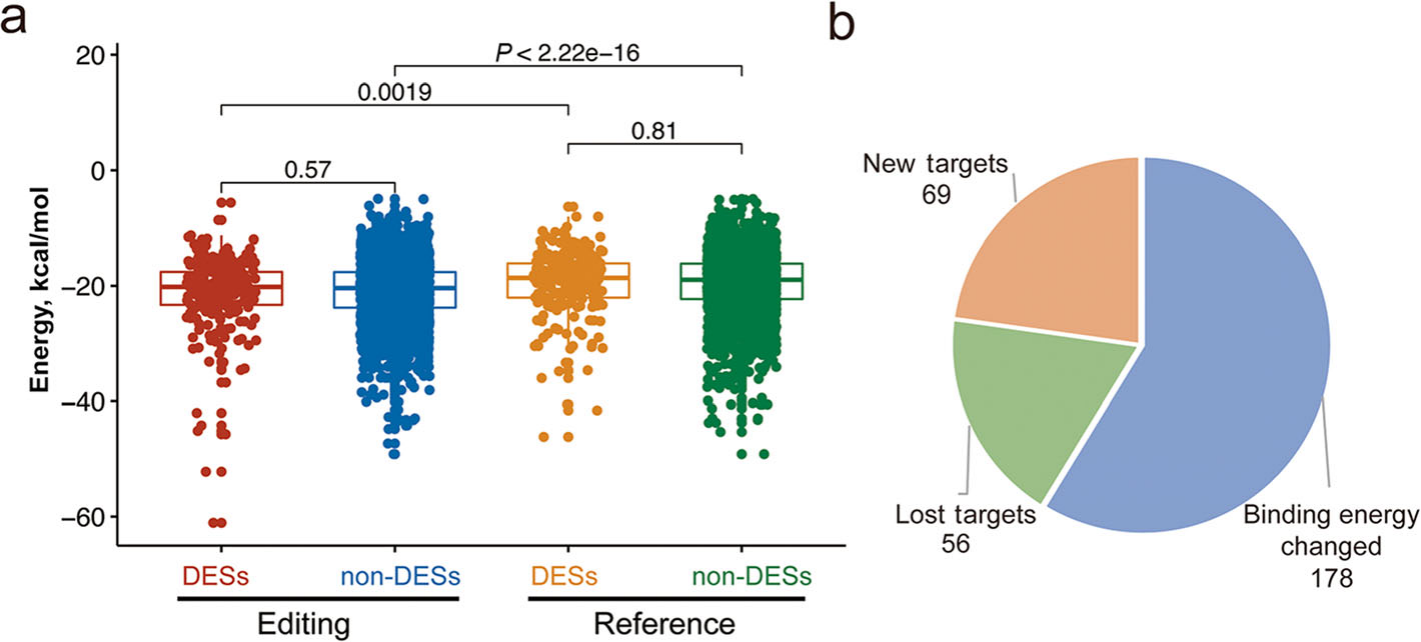
Effects of DESs in the 3′-UTR region on miRNA target binding. **a** Comparing the binding energy of miRNAs with flanking regions of DESs and non-DESs with and without editing in the 3′-UTR region. **b** Pie chart showing the number of miRNA-target pairs affected by DESs in 3′-UTR

To further explore the potential impacts of DESs and their regulatory roles during PG development, the binding energy between miRNA and 3′-UTR regions around the editing sites we computationally predicted. Moreover, it was determined whether RNA editing in 3′-UTR could directly affect miRNA binding. The results showed that the miRNA binding energies of editing sequences was significantly lower than those of reference sequences (Fig. 6a), independent of whether the editing sites were differentially edited during postnatal PG development. Moreover, there was no significant difference in binding energy between the non-DESs and DESs in 3′-UTR (Fig. 6a). A total of 1,284 possible miRNA-target interaction pairs were predicted, which were affected by 143 DESs in 3′-UTR. Among these, 105 sites might change the binding energies of 178 miRNA-target pairs in response to RNA editing. 55 DESs might create new binding sites with the potential to generate 69 new miRNA-target interaction pairs. Moreover, 52 of these DESs led to a disruption of miRNA recognition, which resulted in the loss of 56 possible miRNA-target interactions (Fig. 6b). These results suggest that RNA editing sites might regulate PG development by affecting miRNA binding. For example, the editing type of the DES (chr3:59176514) generated a novel miR-182 binding site in the 3′-UTR of *VAMP5*.

## Discussion

This study identified a total of 47,284 editing events in the porcine PGs at three representative postnatal developing stages (infancy, puberty and adulthood). More than 90% of these editing sites were of the A-to-I type, which is indicative of a canonical ADAR-catalyzed RNA editing event. Consistent with observations in other mammals [11, 12, 35], A-to-I editing predominately occurred in repetitive elements and non-coding regions. To our knowledge, this is the first systematic study on genome-wide RNA editing in PG.

RNA editing can be dynamically regulated by ADAR protein activities. The dynamic expression of RNA editing regulators indicated that A-to-I RNA editing was likely functional during the PG development. Dynamic changes in genome-wide RNA editing during postnatal PG development were demonstrated to be stage-specific. The overall editing level showed a tendency to be higher at Y180 compared with both Y30 and Y300, whereas no positive correlation was observed between the expression of ADARs and the overall editing rate. This suggests a complex regulatory mechanism of A-to-I RNA editing during postnatal PG development.

Differentially edited genes were significantly enriched in vesicle-mediated transport and phosphorylation functions, and similar results were also found in our previous gene expression analysis [22]. Additionally, genes that regulate neuron apoptosis were found to be differentially edited during postnatal PG development, including *PSEN1* and *JAK2*. *PSEN1* mutation has been associated with Alzheimer’s disease [36]. Melatonin can attenuate mitochondrial oxidative damage by activating JAK2/STAT3 signaling [37]. These results suggest that the dynamic change of RNA editing during skeletal muscle development occurred in genes associated with PG physiology. This further suggests that RNA editing plays a critical role in postnatal PG development.

Additionally, genes that underwent simultaneous changes in expression and editing during postnatal PG development were associated with synaptic transmission and ion transport, both of which are essential for the process of communication between neurons. Interestingly, three of these genes (*CACNB2*, *CACNA1A*, and *CACNA1D*) encode voltage-dependent calcium channels, which are involved in a variety of calcium-dependent processes, including cell motility, cell division, and release of hormones or neurotransmitters [38]. These results suggest that the co-transcriptional coordination of gene expression and RNA editing plays an important role during postnatal PG development.

Previous studies suggested that A-to-I RNA editing might disturb the existing miRNA binding or generate novel miRNA binding [5, 13, 39]. The results of the present study showed that the A-to-I editing of 143 DESs might affect the binding ability of miRNA. For example, the editing type of the DES (chr3:59176514) generated novel miR-182 binding site in the 3′-UTR of the vesicle associated membrane protein 5 (VAMP5) gene. miR-182 was reported to regulate the expression of CLOCK, a key component of clock genes, after oxygen-glucose deprivation in primarily cultured pinealocytes [40]. *VAMP5* is involved in neurodegeneration and regulation of mitochondrial processes. Therefore, it can be speculated that the differential RNA editing sites might regulate both the expression and function of gene related to PG development by affecting miRNA binding.

## Conclusion

Overall, this study provides the first comprehensive developmental RNA editome in pig postnatal PG. This new resource is expected to contribute to the understanding of the importance of post-transcriptionally mediated regulation in mammalian postnatal PG development. Although both the function and mechanism of RNA editing in PG remain unknown, these are may be promising targets for further experimental studies of PG development.

## Supplementary information

**Additional file 1 **Summary of the identified A-to-I editing sites in the *Sus scrofa* pineal gland.

## Data Availability

Sequencing data have been deposited to Sequence Read Archive (SRA) at the National Center for Biotechnology Information (NCBI) under accession numbers SRP172576.

## Abbreviations

A-to-I: Adenosine-to-inosine
ADAR: Adenosine deaminase acting on RNA
DEG: Differentially expressed gene
DES: Differentially edited site
FDR: False discovery rate
GO: Gene Ontology
LncRNA: Long non-coding RNA
PG: Pineal gland
RPKM: Reads per kilobase of exon model in a gene per million mapped reads
UTR: Untranslated coding regions
